# Role of AmpR in the High Expression of the Plasmid-Encoded AmpC β-Lactamase CFE-1

**DOI:** 10.1128/mSphere.00192-17

**Published:** 2017-08-09

**Authors:** Ryuichi Nakano, Akiyo Nakano, Hisakazu Yano, Ryoichi Okamoto

**Affiliations:** a Department of Microbiology and Infectious Diseases, Nara Medical University, Kashihara, Nara, Japan; b Department of Microbiology, Kitasato University School of Medicine, Sagamihara, Kanagawa, Japan; JMI Laboratories

**Keywords:** AmpR, plasmid-encoded AmpC β-lactamase, antibiotic resistance, regulation

## Abstract

CFE-1 is a unique plasmid-encoded AmpC β-lactamase with the regulator gene *ampR*. It imparts high resistance to most cephalosporins with constitutive high-level β-lactamase activity. Here, the β-lactamase activities and expression levels of *ampC* with or without *ampR* were investigated. Results suggested that the resistance of CFE-1 to cephalosporins is caused by a substitution in AmpR, in which the Asp at position 135 is modified to Ala to allow the constitutive high-level expression (derepression) of *ampC*.

## Abstract (precis)

CFE-1 is a unique plasmid-encoded AmpC β-lactamase with the regulator gene *ampR*. It imparts high resistance to most cephalosporins with constitutive high-level β-lactamase activity.

## OPINION/HYPOTHESIS

Among the members of the family *Enterobacteriaceae*, the most widespread plasmid-encoded AmpC β-lactamases, such as CMY-2 and CMY-4, are those derived from the chromosomal cephalosporinases of *Citrobacter freundii* (1, 2). The inducible chromosomal AmpC β-lactamase is regulated by the transcriptional regulator *ampR* located upstream of *ampC*. AmpR belongs to the LysR family of transcriptional regulators that typically autorepress their own expression (3, 4). Most of these plasmid-encoded AmpC β-lactamases lack the regulator gene *ampR*, and the insertion sequence IS*Ecp1* is inserted into the AmpR-binding site (2, 5). A *C. freundii ampR* deletion mutant shows a low level of basal *ampC* expression (4, 6); however, high levels of constitutive expression of this mutant by a strong promoter located in IS*Ecp1* lead to resistance to most β-lactams, including oxyimino cephalosporins and some cephamycins (5). A few plasmid-encoded AmpC β-lactamases with *ampR* have been reported; ACT-1 (7) from *Enterobacter cloacae*, DHA-1 (8) from *Morganella morganii*, and CMY-13 (9) from *C. freundii* are inducibly expressed. However, CFE-1, which also carries *ampR* originating from *C. freundii*, is a unique plasmid-encoded β-lactamase that can be constitutively, not inducibly, expressed at high levels to acquire high resistance to most cephalosporins (10). CFE-1 shows remarkably high similarity to the chromosomal gene of *C. freundii* GC3 (10, 11). However, amino acids at positions 221 and 298 of AmpC and at position 135 of AmpR differ from those in *C. freundii* GC3. The constitutively high expression of CFE-1 is predicted to result in a different amino acid sequence at position 135 (Ala for Asp) of AmpR. In this study, we examined the relative expression levels of *ampC* with or without different *ampR* genes and clarified the influence of *ampR* on the expression of *ampC*. The goals of this study were to elucidate the mechanism by which CFE-1 producers acquire high resistance to most β-lactams and to analyze the role of AmpR in the process.

To investigate the effect of AmpR on pKU601, which encodes *bla*_CFE-1_ and *ampR*, we used the constructed plasmids described below. Plasmid pKU602 with loss of function in AmpR was constructed by inserting a GeneJumper kanamycin resistance transposon (Invitrogen Life Technologies, Inc., Carlsbad, CA) into *ampR* of pKU601 (Table 1). The *ampR* gene of pKU601 (encoding AmpR^135A^) or *C. freundii* GC3 (encoding AmpR^135D^ as the wild type) was cloned into the pHSG398 vector (TaKaRa, Japan) as described by Sambrook et al. (12) and named pAmpR135A or pAmpR135D, respectively (Table 1). These plasmids were used to transform *Escherichia coli* GeneHogs and determine the drug susceptibility, β-lactamase activities, and *ampC* expression level of the CFE-1 producers with or without *ampR* as described previously (10, 13, 14).

**TABLE 1:** MICs, β-lactamase activities, and *ampC* expression levels of CFE-1 β-lactamase-producing *E. coli* isolates and their reconstructed transformants

Strain	Genotype	AmpR characteristic	MIC (μg/ml)^a^					β-Lactamase activity (U/mg of protein)^b^	*ampC* mRNA expression^c^
			AMP	CEF	CPD	CTX	CMZ		
*E. coli*(pKU601)	*ampC ampR*	AmpR^135A^	>256	>256	>256	128	128	2.97	1.0
*E. coli*(pKU602)	*ampC ampR*::Km^r^		32	256	32	2	16	0.10	0.17
*E. coli*(pAmpR135 A, pKU602)	*ampC ampR*	AmpR^135A^	>256	>256	128	64	128	2.17	0.82
*E. coli*(pAmpR135 D, pKU602)	*ampC ampR*	AmpR^135D^	8	64	4	0.5	8	0.02	0.06
*E. coli*			1	2	0.25	<0.06	0.25	<0.01	ND^d^

a Antibiotics: AMP, ampicillin; CEF, cephalothin; CPD, cefpodoxime; CTX, cefotaxime; CMZ, cefmetazole.

b β-Lactamase activities are the geometric mean values of three independent cultures. The standard deviations were within 10%.

c Values are relative to the expression of *E. coli*(pKU601), which was assigned a value of 1. The standard deviations were within 10%.

d ND, not done.

The MICs and β-lactamase activities of *E. coli* harboring the constructed plasmids are shown in Table 1. *E. coli* harboring pKU601 is highly resistant to β-lactams and shows higher β-lactamase activity. However, *E. coli* harboring pKU602 (*ampC ampR*::Km^r^) had a significantly decreased MIC of β-lactams and 30-fold lower β-lactamase activity than *E. coli*(pKU601) owing to the loss of function of AmpR. It is suggested that the higher β-lactamase activity of CFE-1 might not depend on the preferentially high hydrolyzing activity against cephalothin, a cephalosporin, but rather on the function of AmpR. Wild-type AmpR usually represses *ampC* expression, resulting in lowered β-lactam MICs, but AmpR^135A^ of pKU601 is thought to be the most likely cause of acquired high resistance to β-lactams. Indeed, *E. coli*(pKU602, pAmpR135A) significantly elevated the MICs of β-lactams and β-lactamase activity by harboring pAmpR135A including AmpR^135A^ at the same level as *E. coli*(pKU601). When pAmpR135D including AmpR^135D^ was introduced along with *E. coli*(pKU602) instead of pAmpR135A, the producer moderately decreased the MICs of β-lactams and the β-lactamase activity compared to those of *E. coli*(pKU602). Thus, AmpR^135D^ might be as effective a repressor of *ampC* expression as wild-type AmpR of *C. freundii*. This implies that *E. coli*(pKU602) showed an effect similar to that of *C. freundii* with the *ampR* deletion mutation, resulting in a slightly higher basal level of *ampC* expression (4, 6). Compared with *E. coli*(pAmpR135D, pKU602), *E. coli*(pAmpR135A, pKU602) showed considerably higher resistance to β-lactams and higher β-lactamase activity. These results indicate that the overproduction of CFE-1 in *E. coli*(pKU601) and *E. coli*(pAmpR135A, pKU602) may depend on the function of AmpR^135A^ as a stronger activator of *ampC*.

To identify the AmpR regulon for high-level expression of *ampC*, we compared the expression profiles of strains harboring AmpR^135A^, AmpR^135D^, or neither. The expression of *ampC* was determined by real-time quantitative reverse transcription-PCR (qRT-PCR). The mRNA expression of *ampC* was normalized to that of the 16S rRNA housekeeping gene, which encodes the ribosomal protein; results are presented as the relative expression of the mRNA compared to that of *E. coli*(pKU601) as described previously (15). qRT-PCR experiments were performed with a LightCycler 480 (Roche, Mannheim, Germany) and SYBR green PCR master mix (Applied Biosystems, CA) in accordance with the manufacturer’s instructions. The primers used were as follows: *ampC*, 5′ CGCTTCCCGCCGTTGAGGTA 3′ and 5′ CCGCCAGTGGAGCCCGTTTTAT 3′; 16S rRNA, 5′ CCAGGGCTACACACGTGCTA 3′ and 5′ TCTCGCGAGGTCGCTTCT 3′ (16). The experiment was performed on three independent occasions, and the final relative expression of *ampC* was determined by averaging the results obtained with the respective transcripts. The coefficient of variation (the standard deviation divided by the mean) among results from different experiments was <10%, and the results are shown in Table 1.

The *ampC* expression of *E. coli*(pKU602) decreased with a decrease in β-lactamase activity, compared to that of *E. coli*(pKU601). Furthermore, *E. coli*(pAmpR135D, pKU602) showed decreased *ampC* expression with AmpR^135D^. Compared with *E. coli*(pAmpR135D, pKU602), *E. coli*(pKU602) showed an effect similar to that of chromosomal *ampC* of *C. freundii* with loss of AmpR function, which decreased its basal expression level (approximately 5-fold higher) (4). This suggests that AmpR^135D^ functions as well as wild-type *C. freundii* AmpR to repress *ampC* expression. However, *E. coli*(pAmpR135A, pKU602) with AmpR^135A^ showed relatively higher expression, which was at the same level as that of *E. coli*(pKU601). *E. coli*(pKU601) and *E. coli*(pAmpR135A, pKU602) exhibited constitutive hyperexpression without induction (data not shown). The data demonstrated that *ampC* expression was increased approximately 14-fold by replacing Asp135 with Ala in AmpR, indicating that AmpR^135A^ activates *ampC* and results in constitutive hyperproduction of the AmpC β-lactamase. The contribution of AmpR to *ampC* expression was analyzed by comparing the relative expression levels obtained with AmpR^135A^ and AmpR^135D^. These results suggested that *E. coli*(pKU601) was highly resistant to β-lactams by hyperproduction of the AmpC β-lactamase, which is regulated by the AmpR activator through substitution of the amino acid at position 135 (Ala for Asp). Some specific point mutations in AmpR may lead to the constitutive hyperexpression of *ampC* and alterations in Gly102 and Asp135 of *C. freundii* (3, 4). An identical mutation at position 135 of AmpR has also been reported as an activator, leading to the constitutive hyperexpression of *ampC* in some species, i.e., Asp135Tyr in *C. freundii* (4), Asp135Asn or Val in *E. cloacae* (17), Asp135Asn in *Pseudomonas aeruginosa* (18), or Asp135Asn in *Stenotrophomonas maltophilia* (19). Thus, these data indicate that the point mutation at position 135 of AmpR plays an important role in the expression of the AmpC β-lactamase; eventually, the substitution of Ala for Asp at position 135 of AmpR facilitates hyperproduction of the AmpC β-lactamase. There is concern about the possibility that a substitution in AmpR of inducible plasmid-encoded AmpC β-lactamases, e.g., the DHA-1 type and the ACT-1 type, might make them highly resistant to β-lactams owing to constitutively high expression of *ampC*.

AmpR has been reported to be a global regulator in *P. aeruginosa*, regulating *ampC* and the expression of several virulence factors through quorum sensing (20–22). DHA-1-producing *Klebsiella pneumoniae* also contains AmpR as a regulator of virulence (23). Furthermore, the acquisition of a plasmid expressing *ampC* alone by *Salmonella* has been associated with a biological cost in the form of reductions in growth rate and virulence (24). It is suspected that the constitutive activator AmpR of CFE-1 could affect the production of virulence factors to provide protection and survival of the bacteria. Further studies are necessary to elucidate the effect of AmpR on mechanisms of virulence and general metabolism in the family *Enterobacteriaceae* with pKU601.

In conclusion, we have definitively shown that the higher resistance of the plasmid-encoded AmpC β-lactamase CFE-1 to β-lactams is dependent on AmpR^135A^, which acts as a constitutive activator of *ampC* expression. This is a unique expression mechanism that has not been reported in any other plasmid-encoded β-lactamase. It is suspected that other AmpC β-lactamases with *ampR*, such as those of the DHA-1 type and the ACT-1 type, would emerge with a mutation in AmpR, resulting in high resistance to β-lactams with constitutively high expression of *ampC*, similar to that in CFE-1.
